# Troponin I at admission in the intensive care unit predicts the need of dialysis in septic patients

**DOI:** 10.1186/s12882-018-1129-5

**Published:** 2018-11-20

**Authors:** Daniel de Almeida Thiengo, Jorge P. Strogoff-de-Matos, Jocemir Ronaldo Lugon, Miguel Luis Graciano

**Affiliations:** 1 Postgraduation Course on Medical Sciences of the Medical School of Universidade Federal Fluminense, Niterói, Rio de Janeiro, Brazil; 2 Nephrology Division, Department of Medicine, Universidade Federal Fluminense, Niterói, RJ Brazil; 3 Centro de Diálise, Hospital Universitário Antônio Pedro, Rua Marques do Parana 303, 2° andar, Niterói, RJ Zip Code 24033-900 Brazil

**Keywords:** Troponin, Acute kidney injury, Biomarker, Sepsis

## Abstract

**Background:**

In a previous study we showed that troponin I (TnI) > 0.42 ng/mL predicted the need of dialysis in a group of 29 septic patients admitted to the intensive care unit (ICU). We aimed to confirm such finding in a larger independent sample.

**Methods:**

All septic patients admitted to an ICU from March 2016 to February 2017 were included if age between 18 and 90 years, onset of sepsis < 24 h, normal left ventricular ejection fraction, and no previous coronary or kidney diseases. TnI was measured on day 1. Patients were followed by 30 days or until death.

**Results:**

A total of 120 patients were included (51% male, 74 ± 13 years old). At ICU admission, 70 patients had TnI > 0.42 ng/mL. These patients had serum creatinine slightly higher (1.66 ± 0.34 vs. 1.32 ± 0.39 mg/dL; *P* <  0.0001) than those with lower TnI and similar urine output (1490 ± 682 vs. 1406 ± 631 mL; *P* = 0.44). At the end of the follow-up period, 70.0% of the patients with lower TnI were alive in comparison with 38.6% of those with higher TnI (*p* = 0.0014). After 30 days, 69.3 and 2.9% of the patients with lower and higher TnI levels remained free of dialysis, respectively (*p* <  0.0001). In a Cox regression model, after adjustment for gender, age, Charlson comorbidity index, serum creatinine, potassium, pH, brain natriuretic peptide and urine output, TnI > 0.42 ng/mL persisted as a strong predictor of dialysis need (hazard ratio 3.48 [95%CI 1.69–7.18]).

**Conclusions:**

TnI levels at ICU admission are a strong independent predictor of dialysis need in sepsis.

## Background

Sepsis is a highly prevalent syndrome and a major cause of death among hospitalized patients. Indeed, this disorder is currently the main cause of death in non-cardiac intensive care units [1, 2]. Sepsis is a clinical syndrome that can be defined as severe infection with a systemic inflammatory response in which dysfunction of different organs and systems may occur [3].

Inflammation is a normal response of the body to infection by microbial agents. However, this process can be inappropriately exacerbated, triggering excessive production of inflammatory mediators and activation of immune cells, ultimately resulting in metabolic anarchy. Such response leads to the involvement of different organs and systems in the so called multiple organ dysfunction syndrome (MODS) [4]. Acute kidney injury (AKI) is commonly found in sepsis and is associated with increased mortality, especially when renal replacement therapy (RRT) is needed [5].

In this context, the identification of early markers of organ dysfunction like AKI would be clinical advantageous in permitting preventive actions that could mitigate the more serious complications of sepsis. Molecules like NGAL, KIM-1 and IL-18 have showed to be able to anticipate AKI [6]. However, such biomarkers are not currently available in the daily clinical practice.

In a previous study, we showed the potential of serum troponin I (TnI) to predict a bad kidney outcome in a group of 29 septic patients admitted to the ICU. Serum TnI levels greater than 0.42 mg/dL in the first day in the ICU accurately anticipated the need of RRT with an estimated area under the curve equal to 0.89 in a ROC analysis [7].

The present study aimed to confirm the value of serum TnI as an early predictor of severe AKI and the need of dialysis in a larger group of patients.

## Methods

### Study design

This is a prospective observational study in which all adult patients admitted with diagnosis of sepsis in the intensive care unit (ICU) of a private hospital at Rio de Janeiro State – Brazil, from March 2016 to February 2017, were eligible to participate. The study was approved by the local ethical committee and an informed consent was signed by the patients or their legal representatives.

Patients were enrolled as long they fulfill the following inclusion criteria: age between 18 and 90 years, less than 24 h since the onset of sepsis symptoms. Those patients with acute coronary syndrome, known previous coronary artery disease or a left ventricular ejection fraction lower than 40% by echocardiography in the first 24 h in the ICU were excluded. Patients with known previous chronic kidney disease, defined as estimated glomerular filtration rate lower than 60 mL/min/1.73 m^2^, known liver disease, AIDS, or recent use of iodinated contrast media, radiotherapy or chemotherapy were also excluded and so did patients who needed RRT on the first day in the ICU.

Patients had the serum TnI levels measured on the admission day to the ICU. Patients who had TnI higher than the pre-established discriminative threshold of 0.42 mg/dL were compared with those with lower TnI levels regarding the need for RRT and mortality.

Patients were followed by 30 days or until death. The primary outcome was RRT initiation and the secondary outcome was all-cause mortality.

### Definitions and procedures

Sepsis was defined as a suspected or confirmed infection in addition to 2 or more points on the Sequential Organ Failure Assessment (SOFA) score according to the Third International Consensus Definitions for Sepsis and Septic Shock (Sepsis-3) [8, 9]. During hospitalization, septic patients were managed according to the recommendations from the Surving Sepsis Campaign [3], regardless of TnI levels.

Urine output and fluid balance were measured every 12 h. APACHE II was calculated in the first 24 h in the ICU. Daily routine laboratory tests included hemoglobin, serum creatinine, blood urea nitrogen (BUN), sodium, potassium, C reactive protein (CRP), brain natriuretic peptide (BNP), and artery pH, HCO_3_^−^ and lactate.

Acute kidney injury was classified according to KDIGO criteria, based on serum creatinine levels and urine output (REF). Decision to initiate RRT was made by the assistant nephrologists, who were unaware of TnI levels, based on their clinical judgement, with no interference of the research team.

### Evaluations

Troponin I measurements were made using the enzyme-linked immunosorbent assay (ELISA). BNP was also measured by ELISA. Other laboratory tests followed standard methods of automated evaluation. Serum TnI was considered normal if lower than 0.05 ng/mL and suspicious of acute coronary syndrome if higher than 1.0 ng/mL.

### Statistical analysis

Results were expressed as mean ± SD if numbers had a normal distribution or, alternatively, as median and interquartile ranges. A comparison of continuous variables throughout the study was performed with Student’s T test in the case of Gaussian distribution or, alternatively, with Mann-Whitney test. Frequencies were compared by Fisher’s exact test. The performance of TnI, serum creatinine, APACHE II and SOFA scores, as a predictor of RRT were evaluated through the calculation of the area under the receiver operating characteristic curve (ROC). The Kaplan-Meier test was used for analysis of survival, and comparison between curves was made by the Log-Rank test. TnI levels at ICU admission and APACHE II after 24 h of admission as predictors of dialysis need were analyzed in multivariate Cox regression models. *P* values < 0.05 were considered significant. The software SPSS, version 18.0 (Chicago, Illinois, USA) was used for the statistical analysis.

## Results

During the recruitment period, 142 patients were initially enrolled in this study, but 22 of them were excluded (thirteen due to left ventricular function in the first 24 h in the ICU lower than 40%, six because of RRT need in the first day in the UCI, one had a diagnosis of AIDS and two withdrew the informed consent). From the 120 patients included in the final analysis, 50.8% were male and the mean age was 74 ± 13 years. At ICU admission, serum creatinine was 1.52 ± 0.40 mg/dL, BUN 30 ± 12 mg/dL, K^+^ 4.4 ± 0.7 mg/dL, pH 7.34 ± 0.07, and TnI 0.64 ± 0.50 mg/mL. Clinical and laboratory characteristics of the patients at ICU admission are shown in Table 1.

**Table 1:** General features of the patients, according to serum troponin I levels at ICU admission

	Overall (*n* = 120)	TnI ≤ 0.42 mg/ml (*n* = 50)	TnI > 0.42 mg/ml (*n* = 70)	*P* value
Age (years)	74 ± 13	71 ± 15	76 ± 10	0.017
Male gender, n (%)	61 (50.8)	18 (36.0)	43 (61.4)	0.009
Troponin I (ng/mL)	0.51 (0.33–1.02)	0.26 (0.02–0.38)	1.0 (0.71–1.03)	–
Serum Cr (mg/dL)	1.52 ± 0.40	1.32 ± 0.39	1.66 ± 0.34	< 0.0001
BUN (mg/dL)	30 ± 12	26 ± 12	32 ± 12	0.0035
Potassium (mEq/L)	4.4 ± 0.7	4.0 ± 0.6	4.7 ± 0.7	< 0.0001
pH	7.34 ± 0.07	7.39 ± 0.05	7.31 ± 0.06	< 0.0001
HCO_3_^−^ (mEq/L)	20.5 ± 3.6	20.4 ± 3.9	20.6 ± 3.3	0.74
Lactate (mmol/L)	2.8 ± 1.6	2.4 ± 1.4	2.5 ± 1.2	0.75
CRP (mg/dL)	7.7 (6.3–10.5)	8.1 (4.2–16.1)	7.0 (6.3–10.5)	0.24
BNP (pg/mL)	63 (36–134)	62 (37–128)	63 (35–145)	0.45
Urine output (mL/24 h)^a^	1461 ± 652	1406 ± 631	1490 ± 682	0.44
Fluid balance (mL/24 h)^a^	+ 1512 ± 428	+ 1539 ± 483	+ 1493 ± 387	0.56
AKI staging by KDIGO^a^				
Stage 0, n (%)	4 (3.3)	4 (8.0)	0 (0.0)	0.028
Stage 1, n (%)	59 (49.2)	32 (64.0)	27 (38.6)	0.009
Stage 2, n (%)	57 (47.5)	14 (28.0)	43 (61.4)	0.0004
Stage 3, n (%)	0 (0.0)	0 (0.0)	0 (0.0)	–

Values are expressed as mean ± S.D. or median (IQR)

*BUN* blood urea nitrogen, *CRP* C reactive protein, *BNP* brain natriuretic peptide, *AKI* acute kidney injury

^a^Assessed in the first 24 h after admission in ICU

On the first day in the ICU, TnI was higher than 0.42 mg/dL, the pre-established cut-off point for high AKI risk, in 70 (58.3%) out of 120 patients. Patients with TnI > 0.42 mg/mL had higher serum creatinine, BUN, potassium and BNP, and lower pH. No difference was found in lactate, HCO_3_^−^, CRP and BNP levels. The urine output and the fluid balance in the first 24 h after admission in ICU were also not different between patients with higher and those with lower TnI levels. No patient was initially classified as AKI stage 3 according to KDIGO criteria, but AKI stage 2 was more frequent among patients with higher TnI levels. No patient was initially classified as AKI stage 3 according to KDIGO criteria, but AKI stage 2 was more frequent among patients with higher TnI levels (Table 1).

The patients with Tn*I* > 0.42 ng/mL showed APACHE II and SOFA score higher than the other patients, but no differences were found in the LVEF or in the Charlson index between this two groups. (Table 2).

**Table 2:** Prognostics features of the patients, according to serum troponin I levels at ICU admission

	Overall (*n* = 120)	TnI ≤ 0.42 mg/ml (*n* = 50)	TnI > 0.42 mg/ml (*n* = 70)	*P* value
APACHE II^a^	16 (10–24)	10 (8–16)	21 (16–25)	< 0.0001
Charlson Index	3 (2–4)	3 (2–3)	3 (2–4)	0.44
SOFA	5.1 ± 2.8	4.3 ± 1.4	6.8 ± 1.1	0.004
LVEF (%)	42.5 ± 4.5	43.2 ± 2.5	42.9 ± 3.1	0.12
Use of norepinephrine^b^	74	32	42	0.08
Mechanical ventilation^b^	47	20	27	0.13
Focus of infection^b^				
Pulmonary	76	30	46	0.07
Urinary	33	15	18	0.11
Abdominal	4	2	2	0.06
Cutaneous	7	3	4	0,18

Values are expressed as mean ± S.D. or median (IQR)

*APACHE II* Acute Physiology and Chronic Health Evaluation II, *SOFA* Sequential Organ Failure Assessment

^a^Assessed in the first 24 h after admission in ICU

^b^Expressed as number of patients

**Fig. 1:**
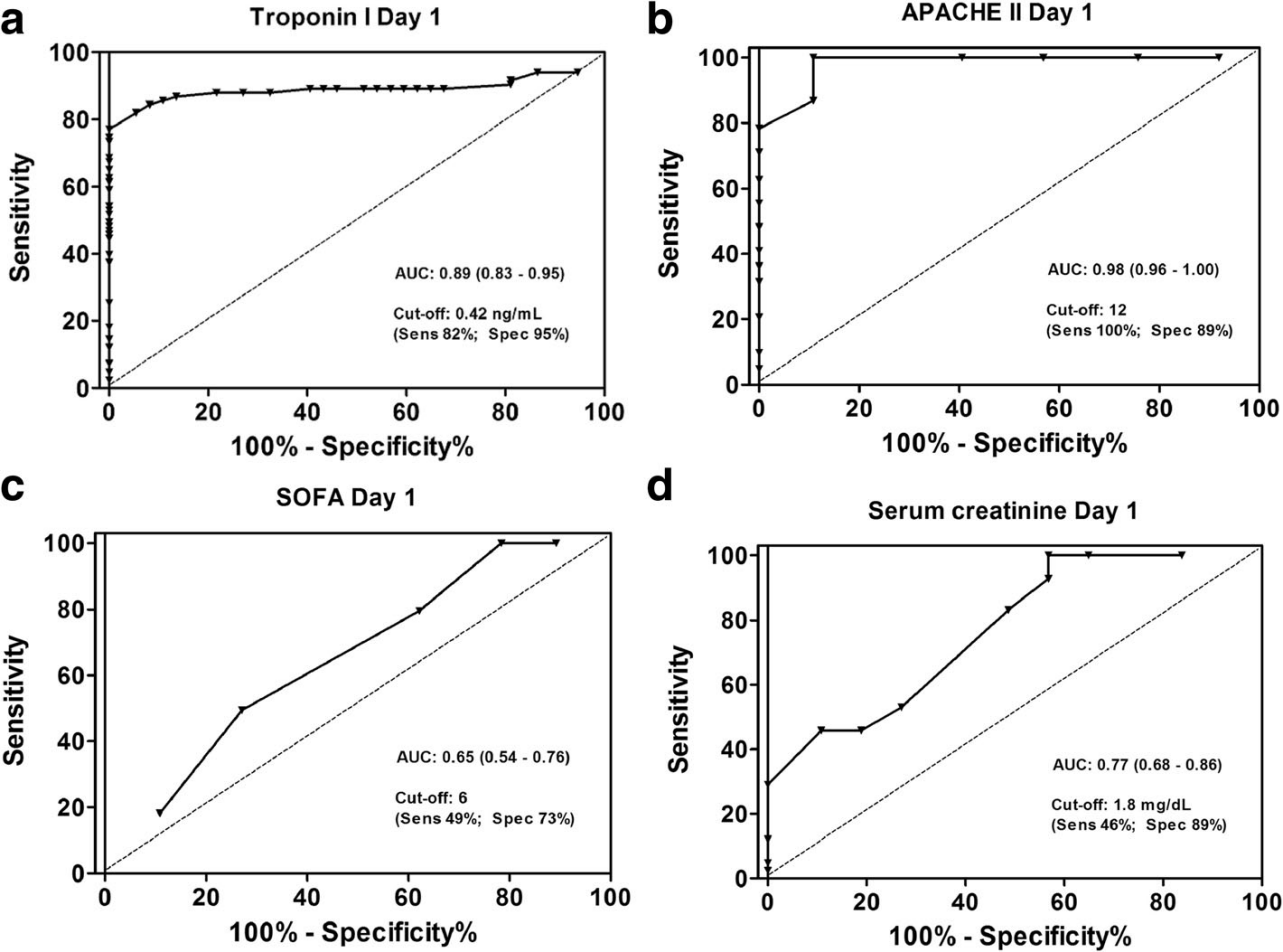
ROC curve of troponin I (**a**) APACHE II (**b**) SOFA (**c**) and serum creatinine (**d**) at ICU for predicting dialysis need

Eighty-three patients underwent RRT during the study, and the time from admission to the UCI until the beginning of dialysis was 8 ± 3 days. ROC analysis showed that TnI levels exhibited an area under curve of 0.89 with the cutoff value of 0.42 ng/mL showing 82% sensitivity and 95% specificity to predict dialysis (Fig. 1a). In a similar analysis, APACHE II calculated in the first 24 h after ICU admission showed a higher accuracy with an area under curve of 0.98, with a 100% sensitivity and 89% specificity for a cutoff of 12 (Fig. 1b). SOFA score and serum creatinine on ICU admission presented poorer performance in predicting dialysis outcome (Fig. 1c and d).

**Fig. 2:**
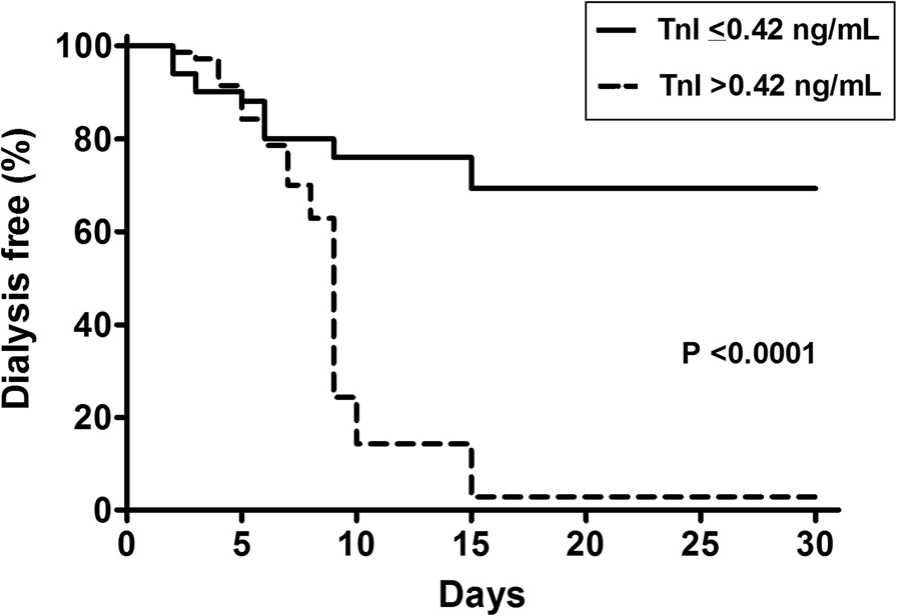
Kaplan-Meier curve for dialysis free survival, according to Troponin I levels at ICU admission

**Fig. 3:**
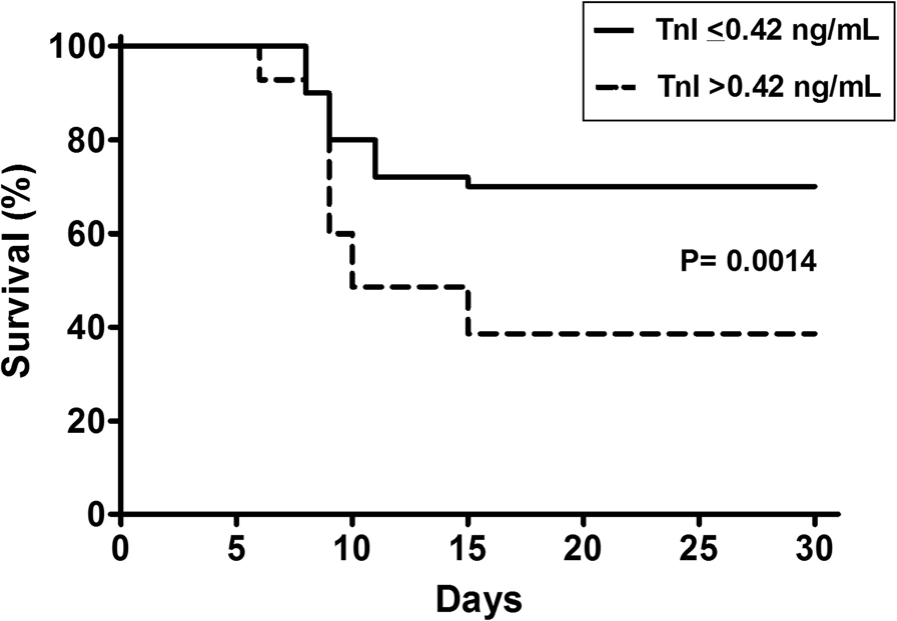
Kaplan-Meier survival analysis, according to Troponin I levels at ICU admission

At the end of the 30-day period of follow-up, 69.3% of the patients with lower TnI at ICU admission remained free of dialysis, whereas 2.9% of patients with higher TnI were RRT free (*p* < 0.0001), Fig. 2. Regarding mortality, at the end of the 30-day period of follow-up, 70.0% of the patients with lower TnI were alive whereas the survival rate for patients with higher TnI was 38.6% (*p* = 0.0014), Fig. 3.

In a Cox regression model, TnI > 0.42 ng/mL at ICU admission, after adjustment for gender, age, serum creatinine, potassium, pH, SOFA, persisted as a strong predictor of dialysis need with a hazard ratio of 3.41 and 95% confidence interval (95%CI) of 1.65 to 7.02, Table 3. In the same model, replacing APACHE II for TnI, it was found a hazard ratio of 1.29 (95%CI 1.22 to 1.38) per each elevation in APACHE II score, Table 4.

**Table 3:** Multivariate analysis of risk of dialysis initiation by a Cox regression model

	Hazard ratio	95% Confidence interval	*P* value
Troponin I > 0.42 ng/mL	3.41	1.65–7.02	0.001
Gender (male)	0.72	0.43–1.22	0.22
Age (year)	0.99	0.97–1.02	0.53
SOFA score D1	1.08	0.87–1.33	0.48
Serum creatinine	2.50	1.25–5.02	0.01
Potassium (mmol/L)	0.95	0.68–1.32	0.48
pH (0.1)	0.34	0.01–1.01	0.056

**Table 4:** Multivariate analysis of risk of dialysis initiation by a Cox regression model

	Hazard ratio	95% Confidence interval	*P* value
APACHE II	1.25	1.21–1.37	< 0.001
Gender (male)	0.84	0.48–1.49	0.56
Age (year)	1.01	0.99–1.04	0.34
SOFA score D1	1.09	0.87–1.36	0.45
Serum creatinine	0.79	0.36–1.70	0.54
Potassium (mmol/L)	1.21	0.82–1.78	0.33
pH (0.1)	0.08	0.01–10.08	0.31

## Discussion

In a previous analysis with a sample of only 29 septic patients we showed that serum TnI levels above 0.42 ng/mL at ICU admission had an accuracy rate higher than 80% to predict dialysis [7]. The present study involving a larger number of patients not only confirmed the ability of serum TnI to predict the need of dialysis in sepsis at admission to the ICU but also to predict mortality in the same group.

The early prediction of the need for dialysis for septic patient has been the subject of many studies [10–14]. The diagnosis of renal dysfunction increases the mortality in the setting of sepsis, especially when RRT is needed. On the other hand, a delayed beginning of dialysis also increases the risk of death [15, 16].

It should be noted that APACHE II as expected was found to be a strong predictor of outcome. Nonetheless, the finding that a prompt and inexpensive test, like troponin I, is able to share this quality, as a predictor of the need for RRT, would be an extremely welcome novelty. Moreover, APACHE II is cumbersome to measure and is attainable only after 24 h of hospitalization whereas TnI can be easily and readily measured at admission.

It is important to note that higher TnI was able to discriminate most of the patients who eventually received dialysis whereas lower TnI values identified all patients who survived and never underwent dialysis. The source of troponin as well as its pathophysiological significance in sepsis deserve further discussion. It is well known that troponin I is elevated in sepsis [17] and correlates with myocardial damage [18, 19]. It is yet to be proved that O_2_ demand-supply mismatch or microthrombosis do play a role on troponin spill over from cardiomyocytes in sepsis [19, 20]. However, direct cytotoxic assault by the septic *milieu* sounds more reasonable. Indeed, DAMP cascade activated by innate immunity during experimental model of Pneumonia-Related Sepsis is able to trigger cardiac inflammation and damage with concurrent troponin I elevation [21]. Accordingly, purely considering our clinical data, we may speculate that troponin I elevation may reflect the severity of inflammation itself. In the present study, elevated TnI at admission was observed at admission in patients more acidotic in addition to higher severity scores (APACHE II and SOFA).

Alternatively, troponin I elevation might be related to renal insufficiency per se. It is known that cardiac enzymes, particularly troponin I are elevated in renal dysfunction [22, 23]. In the current study, it was also observed that TnI was higher at admission in those patients who already presented some degree of renal dysfunction perceived as patients with higher creatinine values and higher renal injury scores. One may discuss whether TnI in these cases just reflects the fall in glomerular filtration rate, however, one should also not that troponin predict better renal outcome than creatinine at entrance to the ICU. We cannot rule out that both possibilities are concurrent and inflammation/cell damage as well as renal dysfunction play a synergistic role in the observed elevation of TnI at day 1. Perhaps a synergistic effect of the reduced TnI clearance due to AKI and the systemic release of troponin as consequence of cell damage in the sepsis can explain an early elevation of TnI and its ability to predict RRT. Regardless of the pathophysiological mechanisms involved, our findings show that TnI levels at ICU admission may have an important and practical clinical implication and can be used as both an early and reliable biomarker of dialysis need and a strong predictor of death risk in sepsis.

The very definition of AKI has presented a challenge along the years. Accordingly, many efforts have been made to uniform the diagnosis and stratification of AKI using traditional markers like creatinine and urine output. To achieve these goals, the classification of RIFLE and subsequently AKIN and KDIGO were developed. However, these important concepts did not emerge with the purpose of predicting the need of dialysis by AKI patients [24].

Despite those more than needed efforts, the presence of creatinine elevation and reduction of urine output are relatively late events during the course of acute renal injury and represent functional consequences of undetected earlier cellular damage [24]. Not surprisingly, there has been a widespread search for early markers of renal injury. Accordingly, NGAL, KIM-1, IL-18 and several other molecules are considered putative markers of early acute kidney dysfunction [11–14]. All those markers perform fairly well in predicting AKI in timed events such as cardiac surgery and iodinated-contrast media administration. However, in the setting of a more blurred insult to the kidneys in the timeline, as in the case of sepsis, such biomarkers have not yet proven their efficacy [25]. It should be mentioned that those tests are not part of routine analysis in most medical centers. In addition, most of them are expensive and their cut-off is not well defined to support use as clinical tools [26]. More recently, promising markers have been proposed like L-FABP and urine angiotensinogen [27] but again their clinical use should await further validation.

Our study has some limitations. Of note, it was conducted in a single center and the number of enrolled patients was relatively small. However, the current study has also some strengths. Firstly, all the patients were derived from the emergency or surgical rooms and had sepsis diagnosed within the last 24 h representing the typical incident patient with sepsis in the ICU. Moreover, this was a prospective study which evaluated an easy-to-perform test widely available in most health facilities that seems to confer relevant information in daily clinical practice.

## Conclusions

Serum troponin I greater than 0.42 ng/mL on the first day at the admission to the ICU can accurately predict which septic patients will eventually need dialysis support. In addition, higher TnI is also associated with an increased risk of death. However, larger studies are needed to further validate our findings.

## Abbreviations

AKI: Acute Kidney Injury
BNP: brain natriuretic peptide
BUN: urea nitrogen
CRP: C reactive protein (CRP)
ELISA: Enzyme-Linked Immunosorbent Assay
ICU: Intensive Care Unit
MODS: Multiple Organ Dysfunction Syndrome
RRT: Renal Replacement Therapy
SOFA: Sequential Organ Failure Assessment
TnI: Troponin I
